# Comparison of musculoskeletal responses and its variability after long-term spaceflight and prolonged bed rest conditions

**DOI:** 10.1038/s41526-026-00611-2

**Published:** 2026-05-25

**Authors:** Jonas Böcker, Patrick Lau, Uwe Mittag, Wilhelm Bloch, Laurence Vico, Jörn Rittweger

**Affiliations:** 1 Institute of Aerospace Medicine, German Aerospace Center, Cologne, Germany; 2 Institute of Cardiovascular Research and Sport Medicine, German Sport University Cologne, Cologne, Germany; 3 SAINBOISE (SAnté INgéniérie BIOlogie St-Etienne), U1059, INSERM (Institut National de la Santé et de la Recherche Médicale), Université Jean Monnet, Mines Saint-Etienne, Saint-Etienne, France; 4 Department of Pediatrics and Adolescent Medicine, University of Cologne, Cologne, Germany

**Keywords:** Anatomy, Health care, Medical research, Physiology

## Abstract

Despite near-daily exercise, musculoskeletal deterioration occurs following spaceflight, with considerable individual variability. This study quantifies the extent, variability, and recovery of musculoskeletal deterioration after 6-month spaceflight (*n* = 13; all male; age: 34–56 years) and compares these findings to those observed during 2-month bed rest without any countermeasure (*n* = 11; all male; age: 22–39 years). Peripheral quantitative computed tomography (pQCT) scans at 4%, 38%, 66%, and 98% of the relative length of the tibia bone from distal to proximal assessed volumetric bone mineral content (vBMC; all measurement sites) and muscle cross-sectional area (Ar.M; 38% and 66%). After spaceflight, Ar.M decreased by 13.3% (SD 5.0%) at the 38% tibial site and by 12.5% (SD 4.9%) at the 66% tibial site, but fully recovered after three months. Bone loss was observed at all measurement sites (*p* < 0.05) and persisted through 3 months (*p* ≤ 0.02). The muscle loss observed post-flight was approximately doubled compared to that was observed 2 months of bed rest, while bone loss was comparable. In conclusion, most comparisons showed no inhomogeneity in variance, indicating that the variability in musculoskeletal outcomes after spaceflight is well represented by bed rest. In summary, bed rest serves as a valuable analog for understanding the mechanisms promoting muscle and bone deterioration during spaceflight.

## Introduction

Long-term exposure to microgravity results in musculoskeletal adaptation in the human body, primarily affecting the weight-bearing muscles and bones of the lower limb^1–4^. The lack of mechanical loading results in muscle wasting and bone loss^5–8^, which may highlight the importance of mechanical stresses as bone anabolic stimulus^9^.

In spaceflight, the amount of bone loss depends, to a large extent, on mission duration^8,10^, with the mean bone mineral density (BMD) loss at the hip and spine in space amounting to 0.5-1.5% per month^11,12^. In the past 2 decades, ESA and NASA missions in low-earth orbit to the International Space Station (ISS) have typically lasted around six months. To counteract the negative effects for bone, crewmembers have been exercising for around two hours per day for six days a week^6,13,14^. Although the applied musculoskeletal countermeasures have progressively improved over the past decades^15^, musculoskeletal adaptations do still occur^16,17^. However, some subjects seem to be more heavily struck than others, and there seems to be variation in the response to spaceflight even within the same individual^2,12,18–20^. One key factor contributing to within-subject variability may be differences in bone geometry and structure. The epiphysis is primarily composed of trabecular bone with a thin cortical shell, whereas the diaphysis consists predominantly of cortical bone. Trabecular bone exhibits a higher turnover rate than cortical bone^21^. However, the results of previous studies showed contrary results regarding the losses of the specific bone tissues. Bone losses assessed by pQCT in response to experimental bed rest, a ground-based analogue for Space, were more pronounced in cortical bone^22^. In contrast, results of high-resolution pQCT as well as QCT obtained a greater loss of trabecular bone after spaceflight^12,23^. Despite these contrary results, these factors contribute to within-subject variability, as do other factors such as cortical thickness or endocortical circumferences^24^.

Between-subject variability in response to bed rest is not an artefact of measurement variability^25,26^ and occurs despite the high levels of standardization of these clinical trials. Generally, Earth-based analogues appear to effectively mimic the actual responses to spaceflight, particularly in terms of skeletal muscle atrophy and bone loss^7,18,19^.

Quite surprisingly, earlier studies reported that bone loss continues following bed rest and peak around 14 days post bed rest – a pattern that has been consistently reported in experimental bed rest^22,27^ as well as in unilateral limb suspension^28^. To date, it remains unclear whether similarly prolonged bone losses occur in space travelers following their return to Earth.

Therefore, the objectives of this study were to evaluate whether or not post re-entry bone losses occur in space travelers, and further to quantitatively explore between-subject variability in response to spaceflight. More specifically, this work addresses the following questions: 1) Despite extensive use of countermeasures during space missions, does significant muscle and bone loss still occur? 2) In line with the analogy of bed rest, is there also a post re-entry bone loss (PREBL) after return to earth? 3) Are the kinetics of recovery between muscles and bone comparable? 4) How do the musculoskeletal effects of a 6-month spaceflight compare with a 2-month bed rest without countermeasures? 5) Can we identify true between-subject variation and within-subject variation in the musculoskeletal adaptation after spaceflight, and how does this compare to bed rest without countermeasures?

## Methods

### Selected studies

This study aims on the musculoskeletal response to microgravity exposure, its recovery and variability, and compares these results with a bed rest analogue. Therefore, we included data sets from the following ESA studies: “Bone loss and recovery after Space exposure” (EDOS-2) and “Reactive Jumps in a Sledge Jump system as a countermeasure during long-term bed rest” (RSL) study^29,30^. EDOS-2 was the follow-up study of Early Detection of Osteoporosis in Space (EDOS)^12^, which explored bone loss and its recovery in space travelers (principle investigators Laurence Vico and Jörn Rittweger). The RSL study was chosen as the bed rest analogue due to the similarity in CT measurement sites and nearly identical measurement dates^29,30^. We selected the control group from the RSL study, which did not perform any countermeasures, because the intervention group performing countermeasures showed no significant bone loss after 60 days of bed rest and countermeasures^29,31^. Additionally, it has been reported that the musculoskeletal response after 60-90 days of strict bed rest is most comparable to the effect after 6 months of spaceflight^19^.

Both studies were conducted in accordance with the Declaration of Helsinki except for registration in a database and have received approval by the ethical committee of the medical chamber North-Rhine (EDOS-2: approval number 2015116; RSL: approval number 2014105), by the Comité d’Ethique du CHU de Saint-Etienne (EDOS-2: approval number: IRBN252014/CHUSTE) and the ethical committee responsible for Gagarin Cosmonaut Training Center (GCTC) in Moscow as well as by the Human Research Multilateral Review Board of the European Space Agency (ESA).

### Participants

We included data sets of 13 male space travelers, but three out of these 13 participants performed two spaceflights during the project. These participants were included twice, resulting in 16 total datasets, as LeBlanc et al. demonstrated that the skeletal response was similar between two flights performed by the same space traveler with approximately two years between the missions^18^. In our study, there were at least 2.5 years between landing after the first mission and launch of the second mission. Furthermore, the space travelers, who were included twice, were exposed to microgravity less than 6 months during their first spaceflight, which is associated with a more pronounced and faster recovery^32^.

All participants gave written, informed consent before participation in the study.

### Peripheral quantitative computed tomography measurements

Data collection of the EDOS-2 study took place from January 29th, 2015 till April, 29th 2021, at the Yuri Gargarin Training Center (GTC) in Star City, Moscow, Russia and at the Institute of Aerospace Medicine, German Aerospace Center (DLR) in Cologne, Germany. Measurements were taken using a XCT2000 pQCT device at GTC and a XCT3000 at DLR. Both devices had been manufactured by Stratec Medizintechnik (Pforzheim, Germany) and are differing solely by their gantry size. Space travelers were either always scanned at GCT (*n* = 15) or at DLR (*n* = 1), and all bed rest participants were scanned at DLR. Both devices were cross-calibrated via measurements with the European Forearm Phantom, which showed a difference of 1.4% for total bone mineral density (BMD), a difference of 1.2% for trabecular BMD and a difference of 1.6% for cortical BMD. For all three comparisons, the difference in assessing the bone area was less than 1%. In our study we used the volumetric bone mineral content (vBMC, mg/mm).

Space travelers underwent two baseline pQCT measurements of the non-dominant leg prior to their launch (BDC: 90 and 45 days before launch) as well as measurements after landing on day 1 ± one day (R + 1), day 14 ± 3 days (R + 14) and day 90 ± 7 days (R + 90). During their mission, the space travelers performed almost daily exercises. However, the exact exercise protocols were not available and could therefore not be integrated in this work.

The RSL study took place in 2015 and 2016 at the Institute of Aerospace Medicine, German Aerospace Center in Cologne, Germany^30^. As for EDOS-2, exclusively male participants underwent two baseline measurements (BDC: 13 and 3 days before bed rest) and three measurements after re-ambulation on R + 3, R + 14, and R + 90, respectively. Between baseline and re-ambulation the participants underwent 60 days of strict 6° head-down tilt bed rest without undergoing or performing any countermeasure.

Each measurement consisted of four individual measurement sites including 4% site of the tibia (Tib04) at the epiphysis close to the ankle, 38% site of the tibia (Tib38), 66% site of the tibia (Tib66), and 98% site of the tibia (Tib98) at the epiphysis close to the knee, respectively, whereas the number indicates the relative position of the measurement site from distal to proximal of the entire length of the tibia bone. Data was processed with the integrated XCT software in its version 6.20, following the procedures outlined in Rittweger, et al.^33^ and Böcker, et al.,^25^ which were highly standardized over the last decades to ensure accurate measurements. For the scans, the resolution was set to a voxel size of 0.5 mm and for each participant the tibia length was measured before the first scan by measuring the distance between the medial malleolus and the medial knee joint cleft. At the beginning of each measurement, a ScoutView was performed, which provides a sagittal image of the bone structures. The ScoutView from the first measurement was set as the reference measurement; for all subsequent scans, the same measurement site was defined using the ScoutView and the automatic function of the software. For the two distal measurements, the reference line was set on the distal tibia plateau, while for the proximal measurements, the reference line was placed on the proximal tibia plateau. Immediately following each measurement, the region of interest was marked by the operator. Bone and muscle areas were determined using a study- and subject-specific segmentation threshold. The total bone area of each measurement was compared to the result of the reference measurement, and in case the deviation was greater than 20 mm², the measurement was repeated. This ensured that the same measurement site was examined in each measurement. The distinction between cortical and trabecular bone was defined by a segmentation threshold of 650 mg/cm², which proved to be accurate for measuring cortical bone. For all measurement sites the total vBMC (vBMC.tot, mg/mm) as the product of the bone mineral density and the total area (Ar.Tib, mm²) divided by 1000 was analyzed as well as separated by cortical bone mineral content (vBMC.Ct, mg/mm) and trabecular bone mineral content (vBMC.Tb, mg/mm) at the epiphyseal measurement sites as described elsewhere^25,34^. Additionally, at the diaphyseal tibia sites (Tib38, Tib66), the muscle cross-sectional area (Ar.M, mm²) of the calf muscles was obtained. The acronyms are in accordance with the previous published work by Rittweger et al.,^33^ who referenced the recommendations for high-resolution pQCT by the ASBMR. A further detailed description of the pQCT data processing is available elsewhere^25,33^.

### Statistical analyses

All statistical computations were performed using R in its version 4.3.2 (www.r-project.org) and RStudio in its version 2023.03.01 (Posit Software, Boston, USA). The underlying code for this study is not publicly available but may be made available to qualified researchers on reasonable request from the corresponding author. The data analysis was closely related to previous published work by Böcker et al.^25^.

For quantifying the musculoskeletal adaptations after spaceflight or bed rest, we calculated the individual percent change *pc*_*i*_ as the relative change of the averaged individual baseline vBMC (vBMC.tot, vBMC.Ct, vBMC.Tb, Ar.Tib) or Ar.M to each post measurement (R + 1/R + 3, R + 14, R + 90).

To detect any significant differences between the several measurement dates and to test for the occurrence of PREBL, an ANOVA for repeated measures for the absolute values of vBMC.tot, vBMC.Ct, vBMC.Tb, Ar.Tib and Ar.M using the aov-R-function was performed. In case of any significant differences, a two-sided pairwise t-test with post-hoc Bonferroni adjustment was computed (R-function: “pairwise.t.test”). Requirements for ANOVA (normally distributed data and variance homogeneity) were checked by a Shapiro test (R-function “shapiro.test”) and F test (R-function “var.test”). Where these were violated, we performed a Kruskal-Wallis test (R-function “kruskal.test”) followed by a pairwise Wilcoxon test with post-hoc Bonferroni adjustment (R-function “pairwise.wilcox.test”).

To compare the changes between space travelers and bed rest participants, a two-sided, non-paired t-test (R-function “t.test”) of the individual change scores (absolute values POST – PRE) was computed and in case of no normal distribution or variance inhomogeneity, a two-sided, non-paired Wilcoxon test (R-function “wilcox.test”) was conducted. To assess the influence of age on muscle and bone loss and its recovery, a linear regression (R-function “lm”) was performed with loss/recovery as the dependent variable and age as the independent variable. Both loss and recovery were defined as change scores (loss: R + 1/R + 3 − BDC; recovery: R + 90 − R + 1/R + 3).

For detection of any differences in the overall variability, we used Levene´s test (R-function “leveneTest” of the car-package in its version 3.1-2) for comparing the variances of the individual change scores for the two conditions. More specific, we calculated measurement uncertainty *U*_*Meas*_, observed uncertainty *U*_*Obs*_ and uncertainty of individual response *U*_*IR*_ as described by Böcker et al.^25^. We defined *U*_*Obs*_ as the variance of *pc*_*i*_, whereas *U*_*Obs*_ is the sum of *U*_*Meas*_ and *U*_*IR*_.

Finally, we identified between-subject variation (BSV) as any individual percent change *pc* that exceeded the 95%-confidence interval defined by the group-site mean values ± 1.96 ∙ *U*_*Meas*_. Thus, we defined BSV as a true variation by differentiation between the individual *pc* and the measurement error. Finally, we analyzed the correlation of *pc* at several measurement sites (Pearson´s correlation coefficient; R-function “cor.test”). In case of any significant correlation, the adaptations were similar for the observed measurement sites. But in case there was no significant correlation, this would indicate the occurrence of within-subject variation (WSV).

## Results

### Participants

The median of mission duration for the space travelers was 178 days, ranging from 165 to 340 days for the space travelers. Only one space traveler had a mission duration which lasted 304 days, and he did not experience the greatest loss of muscle and bone at R + 1. All other mission durations were 205 days or less. The mean age at launch was 47.1 (SD 5.9) years. For bed rest, 11 male participants of the control group of the RSL study were included in the analysis, which underwent 60 days of head-down tilt bed rest without any countermeasures. The mean age was 28.3 (SD 5.5) years.

In total, we included data sets of 13 space travelers in the analysis, even though only 12 data sets were available for the proximal measurement (Tib66, Tib98) sites as one space traveler’s calf was not fitting into the XCT2000 gantry. In the bed rest group, all measurements at the different measurement sites could be included in the analysis for all participants.

### Muscle and bone changes after spaceflight, and their recovery

**Fig. 1:**
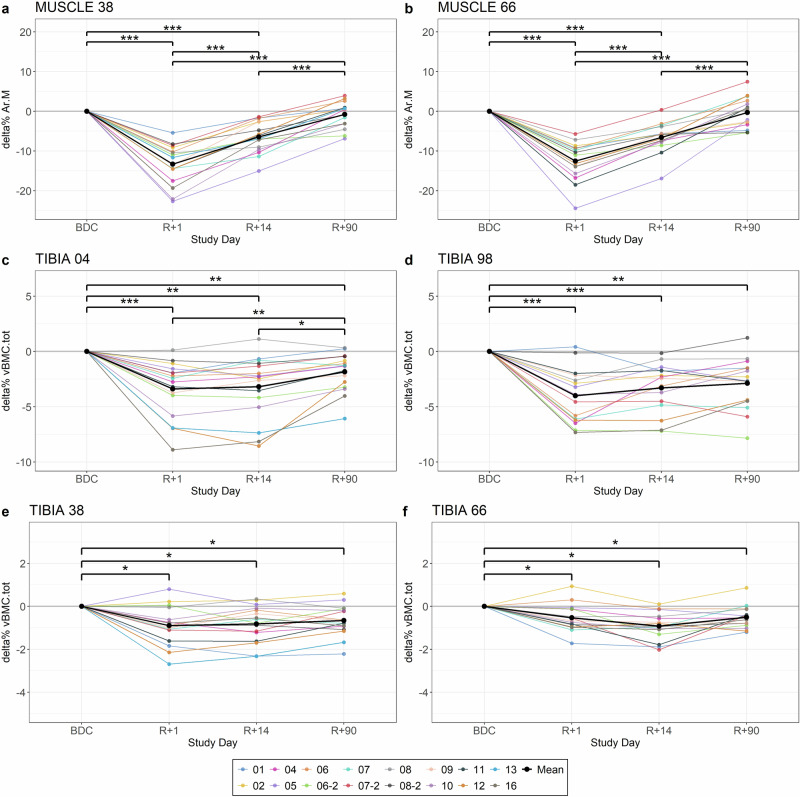
Percent change of Ar.M and vBMC.tot from baseline (BDC) to R + 1, R + 14, and R + 90 after spaceflight, respectively. **a** delta% of Ar.M_Tib38_, **b** delta% of Ar.M_Tib66_, **c** delta% of vBMC.tot_Tib04_, **d** delta% vBMC.tot_Tib98_, **e** delta% of vBMC.tot_Tib38_, **f** delta% of vBMC.tot_Tib66_. “−02” in the legend indicates the results of the second mission of the respective space traveler. These specific results are also listed separately in Fig. 2. The color indicates the different space travelers; black is the mean %delta. * significant difference with *p* < 0.05; ** significant difference with *p* < 0.01; *** significant difference with *p* < 0.001.

After spaceflight, Ar.M_Tib38_ was reduced by -13.3% (SD 5.0%) at R + 1, ranging from −22.7% to −5.4% between different space travelers. Changes for Ar.M_Tib66_ were almost similar (−12.5% [SD 4.9%]). Recovery was also comparable between both muscle sites (Table 1, Fig. 1a, b), leading to full recovery at R + 90 (*p* = 1.00, Supplementary Table 1).

**Table 1:** Muscle and bone changes after spaceflight and bed rest

	Spaceflight			Bed Rest			Space vs. Bed Rest		
Site	BDC – R + 1	BDC – R + 14	BDC – R + 90	BDC – R + 3	BDC – R + 14	BDC – R + 90	BDC – R + 1/R + 3	BDC – R + 14	BDC – R + 90
Ar.M_Tib38_	−13.3 ± 5.0 (−22.7; −5.4)*	−6.5 ± 4.0 (−15.0; −1.4)*	−0.8 ± 3.2 (−6.9; 4.0)	−6.2 ± 4.0 (−12.9; −0.9)*	−3.5 ± 5.2 (−9.5; 7.8)	−0.4 ± 2.4 (−5.4; 2.2)	< 0.001*	0.04*	0.74
Ar.M_Tib66_	−12.5 ± 4.9 (−24.4; −5.7)*	−6.6 ± 3.9 (−16.9; 0.4)*	−0.3 ± 3.9 (−5.4; 7.5)	−7.9 ± 2.7 (−12.0; −2.6)*	−3.7 ± 3.8 (−8.8; 2.4)	2.1 ± 2.5 (−1.4; 5.1)	0.01^a*^	0.07	0.12
vBMC.tot_Tib04_	−3.4 ± 2.5 (−8.9; 0.1)*	−3.2 ± 2.8 (−8.6; 1.1)*	−1.8 ± 1.7 (−6.1; 0.3)*	−2.2 ± 1.4 (−4.8; −0.3)*	−2.4 ± 1.7 (−5.5; −0.8)*	−0.9 ± 1.0 (−3.0; 0.2)*	0.13^a^	0.48^a^	0.07^a^
vBMC.Ct_Tib04_	−29.8 ± 22.9 (−71.0; 1.7)*	−26.5 ± 21.6 (−67.1; 5.3)*	−19.3 ± 17.3 (−47.6; 7.7)	−13.6 ± 18.3 (−34.0; 17.5)	−14.1 ± 17.2 (−46.1; 8.9)	−5.2 ± 15.4 (−24.6; 25.6)	0.21^a^	0.19^a^	0.10^a^
vBMC.Tb_Tib04_	3.3 ± 7.2 (−4.7; 18.3)	2.3 ± 9.2 (−19.7; 17.4)	2.3 ± 8.3 (−13.7; 22.1)	1.9 ± 11.0 (−15.2; 31.0)	3.4 ± 14.8 (−8.2; 47.0)	0.8 ± 10.2 (−19.8; 25.1)	0.48	0.51^a^	0.39^a^
vBMC.tot_Tib38_	−0.9 ± 0.9 (−2.7; 0.8)*	−0.8 ± 0.9 (−2.3; 0.3)*	−0.7 ± 0.7 (−2.2; 0.6)*	−0.7 ± 1.1 (−3.3; 0.7)	−0.7 ± 1.1 (−3.4; 0.1)	0.0 ± 0.3 (−0.6; 0.5)	0.48	0.48^a^	0.003^a*^
vBMC.Ct_Tib38_	−0.9 ± 1.0 (−3.1; 0.7)*	−0.9 ± 0.9 (−2.3; 0.5)*	−0.7 ± 0.7 (−2.3; 0.7)*	−0.9 ± 1.3 (−3.8; 0.7)	−0.9 ± 1.2 (−3.8; 0.2)	−0.1 ± 0.3 (−0.8; 0.4)	0.80	0.68^a^	0.007^a*^
vBMC.tot_Tib66_	−0.5 ± 0.6 (−1.7; 0.9)*	−0.9 ± 0.6 (−2.0; 0.1)*	−0.5 ± 0.5 (−1.2; 0.9)*	−0.5 ± 0.4 (−1.0; 0.0)*	−0.6 ± 0.5 (−1.6; −0.1)*	−0.3 ± 0.3 (−0.8; 0.3)	0.41^a^	0.15a	0.18
vBMC.Ct_Tib66_	−0.5 ± 0.7 (−1.6; 1.2)	−0.9 ± 0.7 (−2.0; 0.4)*	−0.5 ± 0.5 (−1.3; 0.9)*	−0.5 ± 0.4 (−1.0; 0.2)*	−0.7 ± 0.6 (−1.5; 0.4)*	−0.3 ± 0.6 (−1.4; 0.4)	0.86	0.30	0.34
vBMC.tot_Tib98_	−4.0 ± 2.5 (−7.3; 0.4)*	−3.3 ± 2.2 (−7.2; −0.2)*	−2.9 ± 2.3 (−7.8; 1.2)*	−2.0 ± 1.6 (−4.3; 0.3)*	−2.3 ± 1.6 (−4.5; 0.2)*	−1.1 ± 1.0(−3.3; 0.6)	0.01*	0.17	0.03^a*^
vBMC.Ct_Tib98_	−3.5 ± 26.9 (−32.0; 83.9)	−4.0 ± 22.6 (−29.3; 67.2)	−5.1 ± 19.8 (−46.3; 49.6)	−3.4 ± 24.7 (−53.7; 31.1)	−6.9 ± 25.8 (−54.2; 36.3)	−4.6 ± 14.1 (−20.1; 20.3)	0.57^a^	0.32	0.22^a^
vBMC.Tb_Tib98_	−3.8 ± 2.7 (−7.9; 2.4)*	−3.1 ± 2.2 (−7.3; 0.0)*	−2.7 ± 2.2 (−6.9; 1.3)*	−2.0 ± 1.4 (−3.9; 0.2)*	−2.2 ± 1.4 (−4.5; 0.0)*	−1.1 ± 0.9 (−2.8; 0.4)*	0.05^a^	0.20	0.04^a*^

On the left side, mean values of *pc* [%] after spaceflight with standard deviations. In brackets maximum and minimum percent change. In the middle, mean values of *pc* [%] after bed rest with standard deviations. In brackets maximum and minimum percent change. * indicates significant differences from the specific measurement dates to baseline. In the right column, the results (*p*-values) of non-paired t-test comparing absolute bone/muscle loss using Change Scores (absolute values Post – Pre) of Spaceflight and Bed Rest are shown.

^a^indicates that Wilcoxon test was performed as pc for Spaceflight or Bed Rest was not normally distributed or there was no homogeneity of variances between Spaceflight and Bed Rest. As previous, * indicates significant differences.

Bone losses were detected at R + 1 for all bone sites (all *p* < 0.05 for vBMC.tot), showing changes by -3.4% (SD 2.5%) for vBMC.tot_Tib04_, by −0.9% (SD 0.9%) for vBMC.tot_Tib38_, by −0.5% (SD 0.6%) for vBMC.tot_Tib66_, and by -4.0% (SD 2.5%) for vBMC.tot_Tib98_ (Fig. 1c, d, e, f, Table 1). These losses were all still significant at R + 90 (all *p* ≤ 0.02; Supplementary Table 2) and amounted to −1.8% (SD 1.7%) for vBMC.tot_Tib04_, to −0.7% (SD 0.7%) for vBMC.tot_Tib38_, to -0.5% (SD 0.5%) for vBMC.tot_Tib66_, and to −2.9% (SD 2.3%) for vBMC.tot_Tib98_ (Table 1). For vBMC.Ct, there were significant losses at Tib04 (*p* = 0.002) and Tib38 (*p* = 0.02), but not at Tib66 (*p* = 0.08) and Tib98 (p = 0.21) at R + 1. With regard to the epiphyseal trabecular compartment, we observed a drop in vBMC.Tb_Tib98_ by -3.5% (SD 26.9%) (*p* < 0.001), but no change in vBMC.Tb_Tib04_ by +3.3% (SD 7.2%) (*p* = 1.00; Supplementary Table 2) at R + 1.

The analysis of Ar.Tib showed no significant effects (all p ≥ 0.09) for all bone sites at the different measurement dates after spaceflight (Supplementary Fig. 1).

**Fig. 2:**
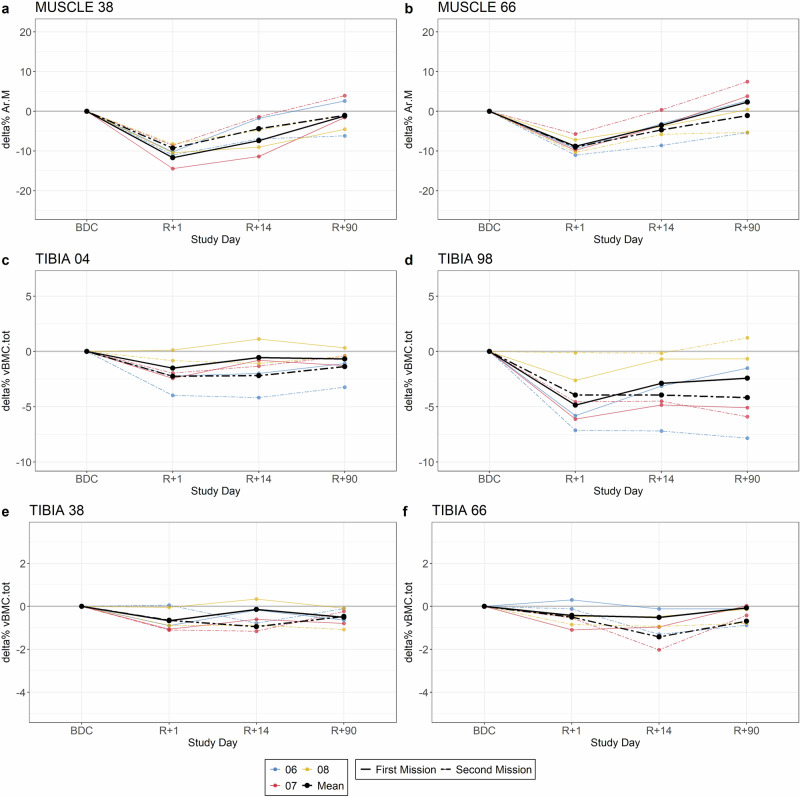
Percent change of Ar.M and vBMC.tot for the space travelers, who went for two space missions. Data from baseline (BDC) to R + 1, R + 14, and R + 90, respectively. **a** delta% of Ar.M_Tib38_, **b** delta% of Ar.M_Tib66_, **c** delta% of vBMC.tot_Tib04_, **d** delta% vBMC.tot_Tib98_, **e** delta% of vBMC.tot_Tib38_, **f** delta% of vBMC.tot_Tib66_.The color indicates the different space travelers; black is the mean %delta. The line type indicates first or second mission.

In examining the subset of space travelers who performed two missions and were therefore represented twice in the study, a clear differentiation can be observed between their first and subsequent missions (Fig. 2, Table 2). As the sample size was limited to only three space travelers, no statistical tests were performed. Despite this, the results indicated similar changes in Ar.M for the first (Ar.M_Tib38_: −11.7% [SD 2.4%]; Ar.M_Tib66_: −8.8% [SD 1.4]) and the second mission (Ar.M_Tib38_: −9.3% [SD 1.5%]; Ar.M_Tib66_: −9.0% [SD 2.9]). Furthermore, at R + 90 the remaining change was comparable for Ar.M after both missions. The loss of vBMC.tot at all measurement sites was similar after the missions, but the remaining bone loss was greater at R + 14 (0.8% to 1.7% greater) and R + 90 (0.6% to 1.8% greater) at all measurement sites after the second mission. The only exception was vBMC.tot_Tib38_ at R + 90, where the remaining changes were comparable.

**Table 2:** Mean values of percent change [%] with standard deviations of the space traveler, who were included twice in the study due to two separate missions (*n* = 3)

Site	Ar.M_Tib38_	Ar.M_Tib66_	vBMC.tot_Tib04_	vBMC.tot_Tib38_	vBMC.tot_Tib66_	vBMC.tot_Tib98_
BDC - R + 1	−11.7 ± 2.4 -9.3 ± 1.5	–8.8 ± 1.4 –9.0 ± 2.9	−1.5 ± 1.4 –2.3 ± 1.6	–0.7 ± 0.5 –0.7 ± 0.6	–0.4 ± 0.7 −0.5 ± 0.4	-4.8 ± 1.9 −3.9 ± 3.5
BDC - R + 14	−7.4 ± 5.0 −4.4 ± 2.8	–3.5 ± 0.4 –4.7 ± 4.6	–0.5 ± 1.6 −2.2 ± 1.7	–0.1 ± 0.5 –0.9 ± 0.2	−0.5 ± 0.4 −1.4 ± 0.6	−2.9 ± 2.1 −3.9 ± 3.6
BDC - R + 90	−1.2 ± 3.6 −1.0 ± 5.1	–2.3 ± 1.7 –1.1 ± 7.4	–0.7 ± 0.9 –1.4 ± 1.6	-0.5 ± 0.4 –0.5 ± 0.5	–0.1 ± 0.1 −0.7 ± 0.2	–2.4 ± 2.4 –4.2 ± 4.8

In the top row, the changes after the first mission, in the bottom row the changes after the second mission.

Given that the tissue within a compartment exhibits similar response, we did not take the individual measurement sites into account while studying the impact of age. Instead we distinguished among the epiphysis (vBMC.tot_Tib04_, vBMC.tot_Tib98_), diaphysis (vBMC.tot_Tib38_, vBMC.tot_Tib66_) and muscle (Ar.M_Tib38_, Ar.M_Tib66_). There was no influence of age on bone loss at the diaphyseal bone sites (space traveler: *p* = 0.24; bed rest: p = 0.89), at the epiphyseal bone sites for bed rest (*p* = 0.24) and for the muscle sites for bed rest (*p* = 0.26). But increased age had a significant influence on bone loss (*p* = 0.03) at the epiphyseal sites as well as muscle atrophy (*p* = 0.049) after spaceflight with older age resulting in greater losses. In the recovery after bed rest, age had no influence, just as for the loss (all *p* ≥ 0.05). However, increased age had an impact on bone recovery at the epiphyseal sites (*p* = 0.03) and an influence on muscle recovery (*p* = 0.007), resulting in decelerated recovery, whereas there was no influence on bone recovery at the diaphyseal sites (*p* = 0.35) after spaceflight.

Turning to the hypothesized occurrence of PREBL, our findings did suggest a trend towards decreases from R + 1 to R + 14 at vBMC.tot_Tib66_ (*p* = 0.06, Fig. 1f), but no such trend was observed at any other site (all *p* ≥ 0.37; Supplementary Table 2). Some space travelers showed measurement site specific individual tendencies to PREBL, e.g., space traveler 06 at Tib04 (Fig. 1c), space traveler 01 at Tib98 (Fig. 1d) and at Tib38 (Fig. 1e), as well as space traveler 06-2 at Tib66 (Fig. 1f).

### Muscle and bone changes after bed rest, and their recovery

Ar.M_Tib38_ showed a mean loss of −6.2% (SD 4.0%) and Ar.M_Tib66_ a mean loss of −7.9% (SD 2.7%) (Table 1, Supplementary Fig. 2), and bed rest-incurred deficits became statistically insignificant at R + 14 (all *p* ≥ 0.06; Supplementary Table 2).

Bed rest-induced bone losses at R + 3 amounted to -2.2% (SD 1.4%, p = 0.001) for vBMC.tot_Tib04_, to -0.5% (SD 0.4%, *p* = 0.008) for vBMC.tot_Tib66_, and to −2.0% (SD 1.6% *p* = 0.01) for vBMC.tot_Tib98_ (Table 1, Supplementary Fig. 2, Supplementary Table 2). The incurred deficit at R + 90 was still a significant for BMC.tot_Tib04_ (*p* = 0.04), but not for any other bone site (Table 1, Supplementary Fig. 2, Supplementary Table 2). For vBMC.Ct and vBMC.Tb, there was no longer any significant difference obtained at R + 90, except for vBMC.Tb_Tib98_ (*p* = 0.03) (Supplementary Fig. 2, Supplementary Table 2).

Even though decreases in vBMC.tot after bed rest from R + 3 to R + 14 were seen at three of four measurement sites (−0.2% for vBMC.tot_Tib04_, −0.1% for vBMC.tot_Tib66_ and -0.3% for vBMC.tot_Tib98_, Table 1), none of these moderate changes was statistically significant (all *p* = 1.00; Supplementary Table 2).

### Spaceflight vs. bed rest responses

**Fig. 3:**
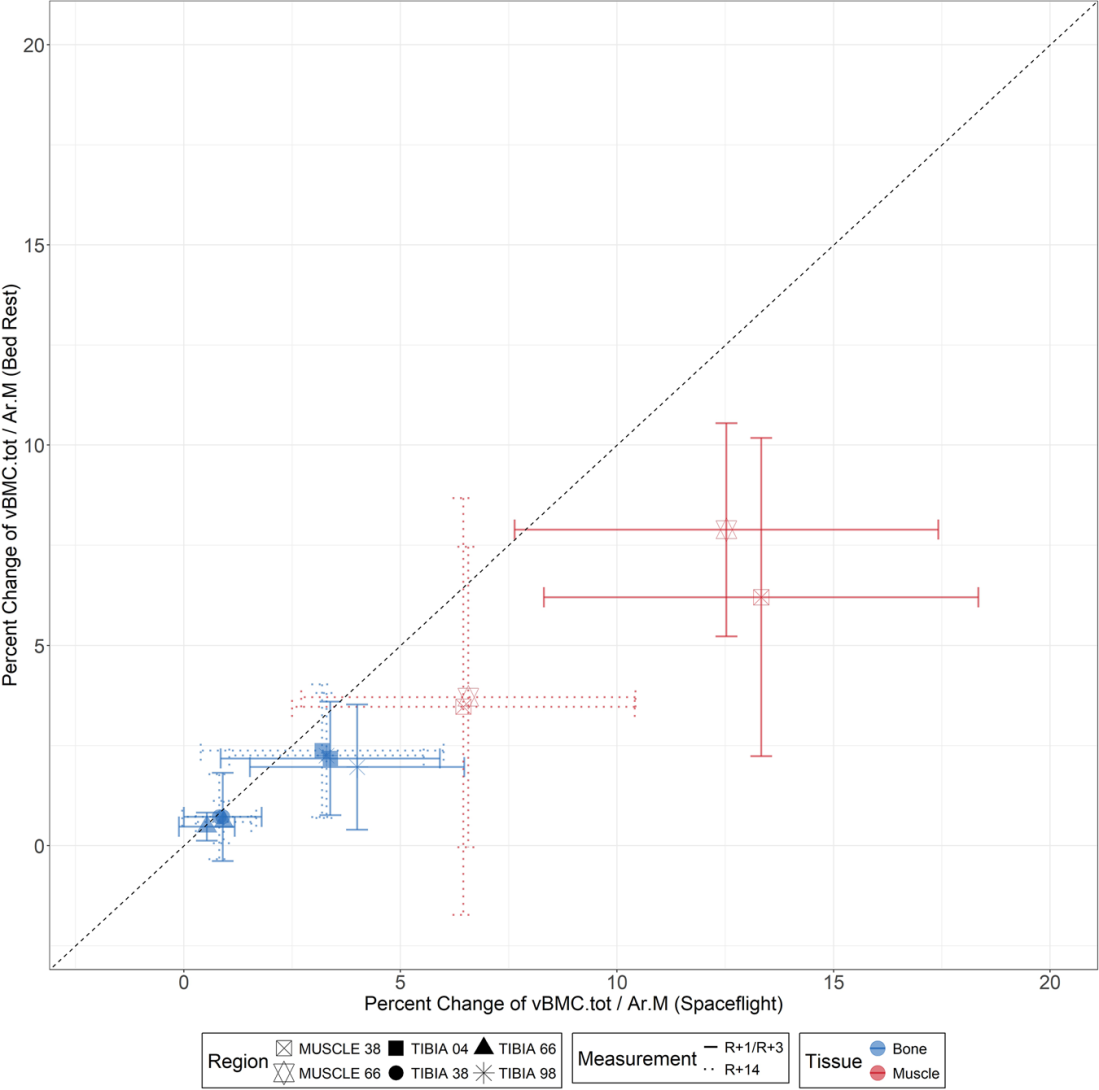
Comparison of spaceflight and bed rest induced musculoskeletal adaptations. Site and group specific percent changes are shown for both conditions. On the x-axis the percent changes after spaceflight, the y-axis showing the percent changes after bed rest. The vertical and horizontal bars show the standard deviation. The different shapes representing the measurement sites, the line type the measurement date and the color the tissue. The black dotted line is the straight line.

When comparing spaceflight and bed rest without countermeasures, changes were generally twice as pronounced in spaceflight as in bed rest with regards to muscle, but they were comparable in relation to bone, particularly at the diaphyseal sites (Fig. 3). More specifically, there were significant differences for change score at the muscle sites at R + 1/R + 3 (Ar.M_Tib38_: *p* < 0.001; Ar.M_Tib66_: *p* = 0.01), but not at R + 14 and R + 90 (except Ar.M_Tib38_ R + 14: *p* = 0.04). Concerning bone tissue, there were only significant moderate differences for vBMC.tot_Tib98_ (p = 0.01) at R + 1/R + 3. The comparison of the recovery showed that there were no differences at R + 14, but differences were present at R + 90. At this point, the change score differed for vBMC.tot_Tib38_, vBMC.Ct_Tib38_, vBMC.tot_Tib98_, and vBMC.Tb_Tib98_, respectively (all *p* ≤ 0.04, Table 1).

### Variability in musculoskeletal response after spaceflight and bed rest

Levene´s test demonstrated only significant inhomogeneity of variances between spaceflight and bed rest for Ar.M_Tib66_ at R + 1/R + 3 (*p* = 0.03) and vBMC.tot_Tib38_ at R + 90 (*p* = 0.01) (Supplementary Table 3). In total, about 5% of the listed *p*-values showed a significant difference.

There were no differences for measurement uncertainty *U*_*Meas*_ between spaceflight and bed rest (all p ≥ 0.10). In both scenarios, *U*_*Meas*_ was greatest for the muscle sites, followed by the epiphyseal sites and the diaphyseal sites (Supplementary Table 4). The observed uncertainty *U*_*Obs*_ as well as the uncertainty of individual response *U*_*IR*_ were more pronounced for muscles than for bones after spaceflight, and were also higher at R + 1 compared to R + 90. When comparing *U*_*Obs*_ and *U*_*IR*_ of spaceflight and bed rest, the results for spaceflight were generally larger, with exception of Ar.M_Tib38_ at R + 14 and vBMC.tot_Tib38_ at R + 1 and R + 14 (Supplementary Tables 5 & 6).

**Fig. 4:**
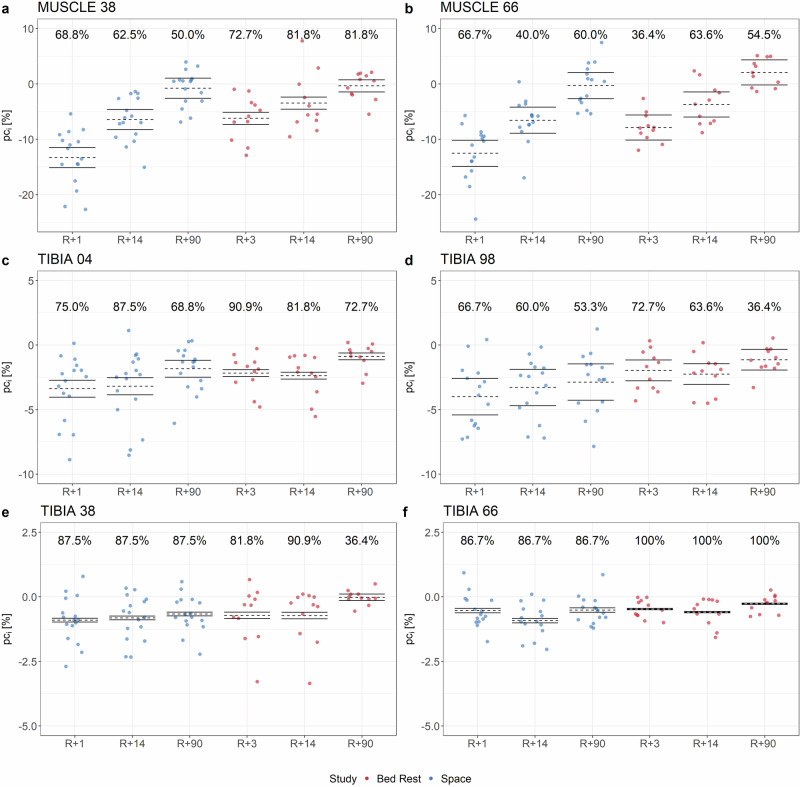
Overview of the between-subject variation (BSV) of spaceflight and bed rest. BSV indicated by exceeding the confidence interval by the vast majority of the individual percent change *pc*_*i*_ in [%] with **a** *pc*_*i*_ of Ar.M_Tib38_, **b** *pc*_*i*_ of Ar.M_Tib66_, **c** *pc*_*i*_ of vBMC.tot_Tib04_, **d** *pc*_*i*_ of vBMC.tot_Tib98_, **e** *pc*_*i*_ of vBMC.tot_Tib38_, **f** *pc*_*i*_ of vBMC.tot_Tib66_ Dotted line presents the mean value, solid lines are upper and lower limit of the confidence interval based on 1.96 ∙ U_Meas_. The color indicates the condition, where blue represents space and red bed rest. Values about each plot represent the relative number of individual percent changes exceeding the confidence interval.

In regard to BSV, it was comparable between spaceflight and bed rest (Fig. 4). For both conditions, the BSV was mostly elevated for the diaphyseal bone measurement sites (vBMC.tot_Tib38_, vBMC.tot_Tib66_; Fig. 4e, f) except vBMC.tot_Tib38_ at R + 90 after bed rest (36.4%). Furthermore, BSV tended to be smaller for the muscles than for the bone sites (Fig. 4).

WSV was found to be comparable between spaceflight and bed rest (Table 3). Significant correlations were observed for Ar.M from baseline to R + 1 (spaceflight: *p* < 0.001; bed rest: *p* = 0.007) and R + 14 (spaceflight: 0.007; bed rest: *p* < 0.001) with good to excellent correlations (*r* ≥ 0.63) under both conditions. The comparison of vBMC.tot at the diaphyseal measurement sites showed a significant correlation for spaceflight and bed rest (all *p* ≤ 0.02; *r* ≥ 0.60). For all other significant correlations (all *p* ≤ 0.04), there was at least a good correlation (all *r* ≥ 0.55). Nevertheless, most comparisons had no correlation and therefore demonstrated WSV. For spaceflight, 19 out of 26 showed WSV (73.1%), for bed rest 16 out of 26 (61.5%) (Table 3).

**Table 3:** Results of the Pearson correlation analysis for percent change from pre-mission/pre-bedrest to post-mission/post-bedrest divided by study days and measurement sites (top: *p*-value, bottom: correlation coefficient)

Study	Study Day	Ar.M_Tib38_/Ar.M_Tib66_	vBMC.tot_Tib04_/vBMC.tot_Tib38_	vBMC.Ct_Tib04_/vBMC.tot_Tib38_	vBMC.tot_Tib04_/vBMC.tot_Tib66_	vBMC.Ct_Tib04_/vBMC.tot_Tib66_	vBMC.tot_Tib04_/vBMC.tot_Tib98_	vBMC.Ct_Tib04_/vBMC.Ct_Tib98_	vBMC.Tb_Tib04_/vBMC.Tb_Tib98_	vBMC.tot_Tib38_/vBMC.tot_Tib66_	vBMC.tot_Tib38_/vBMC.tot_Tib98_	vBMC.tot_Tib38_/vBMC.Ct_Tib98_	vBMC.tot_Tib66_/vBMC.tot_Tib98_	vBMC.tot_Tib66_/vBMC.Ct_Tib98_
Space	BDC - R + 1	< 0.001 0.80	0.07 0.46	0.18 0.35	0.26 0.31	0.23 0.33	0.03 0.55	0.14 -0.40	0.006 0.67	0.006 0.68	0.75 -0.09	0.69 0.11	0.38 -0.25	0.83 -0.06
	BDC - R + 14	0.01 0.63	0.21 0.33	0.21 0.33	0.86 0.05	0.41 0.23	0.003 0.70	0.43 -0.22	0.47 0.20	0.002 0.73	0.61 0.14	0.24 0.33	0.40 0.23	0.75 -0.09
Bed Rest	BDC - R + 3	0.007 0.75	0.21 0.41	0.13 0.49	0.16 0.45	0.22 0.40	< 0.001 0.85	0.005 0.78	0.47 0.25	< 0.001 0.87	0.52 0.22	0.23 0.39	0.16 0.45	0.15 0.46
	BDC - R + 14	< 0.001 0.91	0.72 0.12	0.04 0.64	0.46 0.25	0.03 0.66	0.08 0.55	0.18 0.44	0.05 -0.59	< 0.001 0.87	0.009 0.74	0.57 0.19	0.003 0.81	0.32 0.33

Non-significant correlation indicates a within-subject variation (WSV).

## Discussion

The results of the study show that, despite intensive physical training, there was still a significant loss of muscle tissue and bone mass following 180 days of microgravity exposure. Notably, the space traveler who spent 340 days in space did not exhibit the greatest losses. Contrary to our hypothesis, we did not observe an overall protracted bone loss after landing, discarding the occurrence of PREBL. Muscle atrophy experienced by the space travelers was approximately twice as severe as that observed after 60 days of bed rest, while bone loss levels were comparable between the two conditions. Although the overall percentage of muscle loss differed substantially between spaceflight and bed rest, the variability in individual changes was comparable for muscles and bones. Furthermore, BSV and WSV also showed no differences between the two conditions.

Although the space travelers performed almost daily physical training, which has been systematically improved since the beginning of space travel^35^, there was still significant loss of bone (Fig. 1, Table 1), which was caused by a decrease of the bone mineral density as the total bone area did not change (Supplementary Fig. 1). These losses, particularly at the diaphyseal measurement sites, were comparable to the bone degradation observed after 60 days of bed rest without any countermeasures (Fig. 3). In contrast, muscle loss after spaceflight and bed rest differed significantly. Previous bed rest studies showed that muscle loss follows a logarithmic pattern^36^, whereas bone loss follows a linear pattern^22^. However, the duration of bed rest in these studies was longer for muscles than for bones. The results of our study suggest that the greatest bone loss occurs within the first 60 days and slows considerably thereafter, leading to comparable results despite the different durations (approximately 6 months of spaceflight and 60 days of bed rest), which need to be proved in possible future studies. It is important to consider, however, that the space travelers performed countermeasures to maintain musculoskeletal health, whereas the bed rest participants did not. Furthermore, it must be discussed that our results show a 3.3% increase in vBMC.Tb_Tib04_ on average after spaceflight. It should be noted that vBMC.Tb was calculated as the difference between vBMC.tot and vBMC.Cort. Therefore, the calculation of vBMC.Tb does not incorporate the specific bone mineral density of the trabecular structures. This can lead to slight inaccuracies in the calculation of vBMC.Tb, so these results should be interpreted with caution.

If one considers the space traveler with two missions individually, it appears that there is an influence whether a space traveler completed a first or second mission. While the loss of muscle and bone is comparable between the two missions, the recovery of vBMC.tot is decelerated (Fig. 2, Table 2). This might be due to incomplete regeneration after the first mission, as previous studies have indicated that two years were sometimes insufficient for complete bone mass regeneration^16,37^, changes in 3D structures after recovery^38^, changes in muscle-bone crosstalk^9^ or epigenetic changes in bone cells or pathways, which affect the bone cells and its function^39^. A second factor could be the influence of the older age during the second mission; however, our results only show a significant influence of age on regeneration in the epiphysis.

In contrast to the hypothesis that bone degradation would continue after returning to Earth, this study - incorporating data from both spaceflight and bed rest - found no significant bone loss between R + 1 and R + 14. (Fig. 1, Supplementary Table 1). It is important to note that some space travelers exhibited site-specific PREBL; however, none showed a progression of bone loss across all measurement sites. In case of spaceflight, certain factors appear to hinder the prompt and complete recovery of bone tissue after return to Earth. Previous studies have reported that baseline bone mass is often not fully restored even after extended recovery periods^12,16,32^ and that mission durations exceeding a critical threshold of six months may impair recovery capacity^32^. However, in this study, such an effect was not observed in the space traveler who spent 340 days in space. One difference between spaceflight-induced bone loss and that observed during bed rest is exposure to ionizing space radiation. This factor could possibly lead to a reduced bone recovery as it was shown that radiotherapy could reduce bone formation^40^. But there are several more factors, which could influence bone recovery, e.g., fluid intakes, differences in metabolism^10^. Furthermore, there are factors that have an influence but cannot be reproduced in bed rest studies. These include, in addition to the aforementioned radiation, social isolation, reduced sleep quality, and altered nutrition. In general, it is difficult to identify mechanisms that explain the differences in bone recovery between spaceflight and bed rest due to the small number of space travelers. Therefore, the Earth analogue bed rest is usually used to study these processes, resulting in a lack of knowledge when comparing these two conditions^32^.

Additionally, it is recommended to investigate potential factors that may contribute to increased bone resorption. However, it was shown that trabecular bone is more prone to deterioration^41^, which is in contrast to our findings. This discrepancy can be explained by differences in the participant selection as the cited study included two participants (1 female). But other studies obtained that trabecular bone is subject to greater metabolic activity because it has a higher surface area to volume ratio and is therefore more responsive to changes in mechanical stress, nutrition, hormones, etc^9^. Of course, there are other factors that influence bone preservation and recovery, such as the pre-flight individual rate of bone turnover, pre-flight training habits^10^, diet, lifestyle habits, and health status before microgravity exposure^11,42^.

Orwoll et al. stated that only resistive training, a training modulus using greater forces to generate greater strain, is regularly used to prevent bone loss^43^, but treadmill running on the T2 can also generate impacts, resulting in skeletal strains, which elicit osteogenesis. But as high loads on the treadmill generated by the bungee ropes possibly go hand in hand with painful abrasions due to the harness, space travelers often run at lower loads, which results in lower impacts^35^. However, in order to achieve effective skeletal strains stimulating osteogenesis during entire mission duration, novel countermeasure approaches need to be considered. Plyometric training, characterized by a greater rate of loading (e.g. moderate loads at high velocities), was tested as effective in a 2-month bed rest campaign. Low-volume, high-intensity jump training maintained structure and function of three different organ systems at once (bone, muscle and cardiovascular system)^29^. One might wonder, however, whether this type of exercise can be performed in space in a way that ground reaction forces are as effective as they are on Earth.

Comparing muscle wasting between spaceflight and bed rest is not straightforward, given that space missions in this study lasted three times longer than experimental bed rest, and that space travelers exercised daily whilst bed rest participants performed no countermeasure at all. Nevertheless, this study showed that the percent change of Ar.M after spaceflight was about twice as large as after bed rest at the two measurement sites. At this point, it needs to be considered that we only analyzed the lower limb, which is more prone to muscle deterioration compared to the upper limbs^3^. It must be further discussed that pQCT was originally developed to examine bone parameters^44^. Accordingly, pQCT is not the gold standard for determining Ar.M. In comparison to pQCT, magnetic resonance imaging (MRI) offers the possibility of imaging muscles at high resolution and differentiating between individual muscles and different tissue types^44^. Furthermore, MRI does not use ionizing radiation. Dual-energy X-ray absorptiometry (DXA) is also used to determine lean muscle mass, including regionally^44^. While DXA offers the possibility of determining individual slices, its short-term error rate is higher compared to pQCT^22^. pQCT is also preferable to DXA when it comes to determining tissue composition with regard to muscle strength^45^.

However, it should be noted that the measurement dates differed (spaceflight: R + 1; bed rest: R + 3). If one compares spaceflight results with results assessed at day 60 of head-down tilt bed rest, the losses of Ar.M were even greater (Ar.M_Tib38_: -18%; Ar.M_Tib66_: -21%)^25^. But fluid shifts during re-ambulation lead to a return to the initial distribution within 2 h^46^. Therefore, the results of R + 1 (spaceflight) and HDT60 (bed rest) are not comparable, although they are closer in time.

**Fig. 5:**
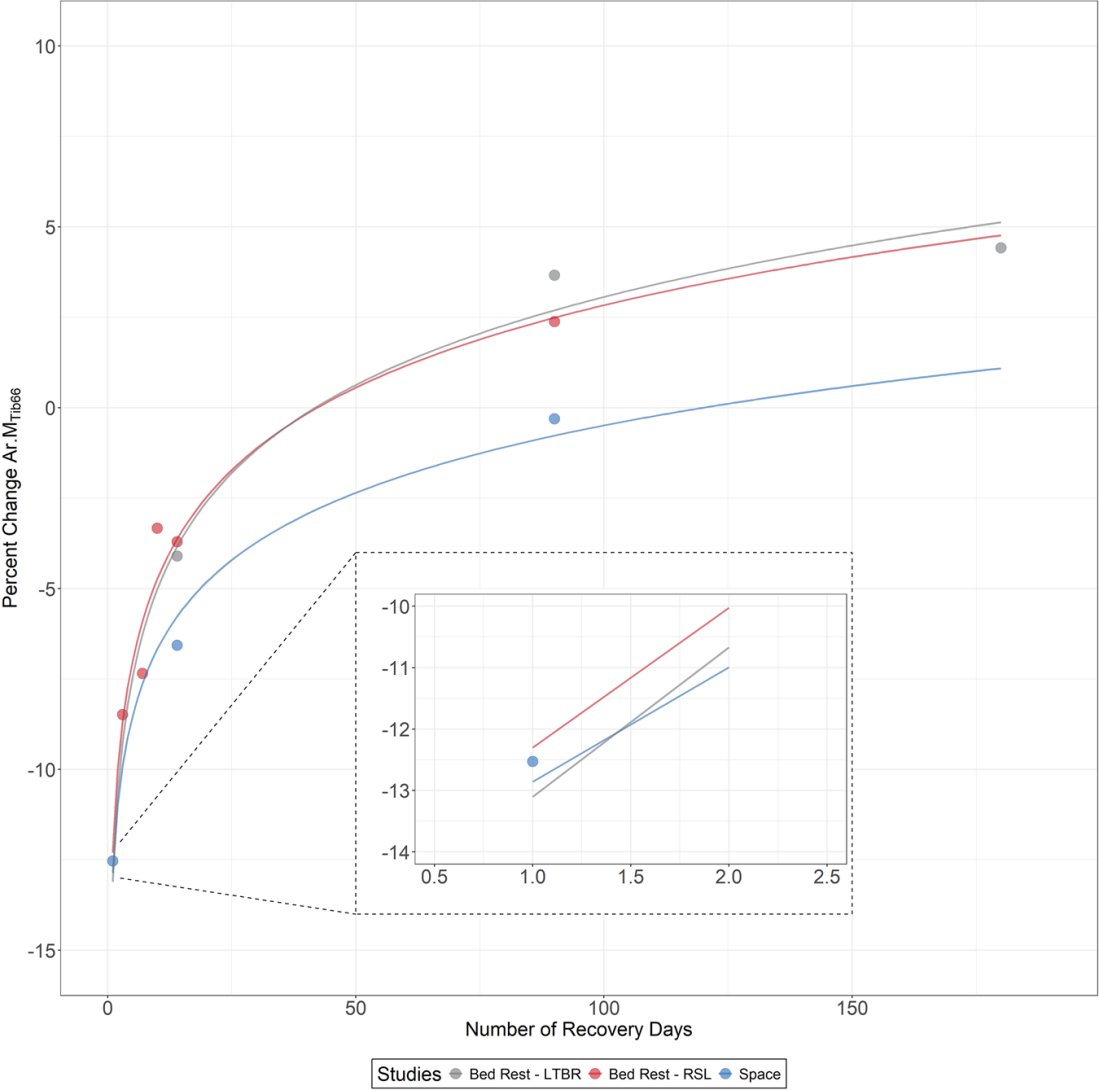
Logarithmic function of muscle recovery after spaceflight or bed rest induced changes in Ar.M_Tib66_. From bed rest studies, this analysis included just the percent changes of the control groups, which underwent bed rest only (LTBR: 90 days of 6° head-down tilt bed rest; RSL: 60 days of 6° head-down tilt bed rest). The estimation shows that the muscle atrophy is lower after 60 days of bed rest compared to 180 days of spaceflight, but muscle atrophy after 90 days of bed rest exceeds the spaceflight amount.

Based on these findings, we aimed to estimate the required bed rest duration to elicit comparable muscle loss to that observed after spaceflight. For this purpose, we used data of the control group without any physical training from the LTBR study (long term bed rest: 90 days of 6° head-down tilt bed rest)^27^, which had a bed rest duration of 90 days. However, the measurement dates after re-ambulation were R + 14, R + 90, and R + 180. Therefore, to make a statement about muscle loss at R + 1, the result for R + 1 had to be estimated. It was previously shown that muscle loss and muscle recovery can be described by a logarithmic function^36,47,48^, thus, we fitted a logarithm function for the available datasets. Figure 5 shows that after 90 days, the muscle loss would be greater, but that strict 6° head-down tilt bed rest of approximately 80 days leads to a comparable loss of Ar.M. as 180 days of microgravity exposure with physical training.

However, the loss of muscle and bone was subject to between-subject differences, represented by a large range, as well as within-subject differences as already shown in Vico et al.^2^ and Lang et al.^7^. As shown in Fig. 4, bone showed a greater BSV than muscle. However, in addition to these general results, the individual results also show clear differences between the space travelers. For example, the space traveler whose mission was significantly longer than all the others showed only slight decreases in vBMC.tot_Tib98_, whereas individual space travelers with significantly shorter missions showed a much greater loss. This could be due to genetic differences that favor a heavy disposition for bone loss. Furthermore, it is likely that the space travelers respond differently to training onboard the ISS (responder vs. non-responder)^49,50^ and have different training discipline. In the view of future deep-space missions, which will last clearly longer than the current missions, methods to identify crew candidates with those kinds of predisposition are mandatory to minimize the risk of musculoskeletal hazard.

Beside all these aspects, it has already been described that just after bed rest there is a large variability in musculoskeletal response between participants^18,25,27^ as well as between different measurement sites within a participant^25,27^. Our results are also confirmed by Sibonga et al. (2020), who showed that there was a great between-subject variation regarding bone resorption markers for space travelers during and after their missions^16^.

Scott et al.^31^ have published that bed rest is a good way to simulate the variability after microgravity exposure and the use of countermeasures. This was also reflected in our results, as the variances of individual adaptation differed only in a few cases between space and bed rest (Supplementary Table 3). The investigated parameters *U*_*Meas*_, *U*_*Obs*_ and *U*_*IR*_^25^ were larger for space (Supplementary Table 4-6), but these results were not significant. Thus, these findings further support the use of bed rest as a valid spaceflight analogue for capturing individual variability. When comparing the bed rest results for *U*_*Meas*_, *U*_*Obs*_ and *U*_*IR*_ with the data from Böcker et al.,^25^ small differences are noticeable. These are due to the fact that different measurement days were used for the calculation in this study to ensure comparability with the space data. Furthermore, we considered BSV and WSV in this work, which were both comparable between spaceflight and bed rest. Naturally, the reasons for between-subject variability (BSV) differ slightly between spaceflight and bed rest, partly due to differences such as the daily physical training performed by space travelers and their generally older age compared to bed rest participants. Nonetheless, the primary factors driving this variability are likely to be shared across both conditions.

Thus, despite the different degree of standardization, the bed rest analogue is also suitable for illustrating the variability after spaceflight. This offers the opportunity to work out these further factors that favor variability^19,51^, which is very important for the future selection of space travelers^2,51^.

The main limitation is the different timing of data collection after spaceflight or bed rest (R + 1 vs. R + 3). The first two days naturally have a clear influence on the adaptations of the muscles. However, Fig. 3 shows that despite the different time points, bone loss is very comparable. In this context, it needs to be mentioned that despite all precautions, there is still a minor risk of measurement error for BMC in case of slightly shifting the region of interest from measurement date to measurement date. Furthermore, the recovery data allowed us to take an additional approach to describe muscle loss. In relation to the calculations of vBMC, it should also be mentioned that vBMC.Tb was calculated as the difference between vBMC.tot and vBMC.Cort. This could potentially introduce minor inaccuracies for vBMC.Tb, thus, the results of vBMC.Tb need to be interpreted carefully. Another factor that must be taken into account is the difference between the groups in terms of age. However, it will be almost impossible to compare space travelers and bed rest participants who are at the same age. Space travelers are a small group of people who, due to their previous education, are older per se than participants in bed rest studies, who tend to be younger, usually due to their professional or private situation. To further refute this aspect, we analyzed the influence of the age of the two groups on bone loss and muscle atrophy as well as its recovery. What also needs to be considered when interpreting the results is the fact that three of the space travelers were included twice. We considered these data as separate data sets, as adaptations in subsequent missions were the same^18^, but it cannot be guaranteed that the original bone architecture and mass were fully restored^12,32^. Regarding limitations of the statistics, it must be mentioned that with the large number of tests performed to compare variance of change scores, significant results could occur by chance (Type I error). We therefore interpreted the results cautiously, and they suggest that spaceflight and bed rest cause very similar variabilities of musculoskeletal adaptations. Another limitation is the lack of training documentation for the space travelers. We also had no information regarding further countermeasures and no overview of the diet, all influencing the adaptations and the variability. All these parameters were highly standardized during the bed rest. Furthermore, bed rest studies do not offer the possibility of mapping all factors that have an influence on musculoskeletal adaptations during spaceflight, such as radiation. However, it has been shown in this study and in previous studies that space missions of 6 months can still be compared with bed rest studies with a duration of 60 to 90 days^19,27,31^.

In conclusion, this study demonstrates that despite improved countermeasures and almost daily exercising, a significant decrease in muscle and bone mass still occurred after 6 months of exposure to microgravity. Contrary to the experience from some previous bed rest studies, bone loss did not continue for all space travelers after the return to Earth, thus, showing no PREBL. Only some individuals experienced continued bone loss after their missions. This study also revealed that skeletal adaptations in the lower limb following a six-month mission could be effectively modeled by 60 days of bed rest without countermeasures, whereas muscle changes required approximately 80 days of bed rest for comparable effects. In addition to the observed losses in muscle and bone mass, both between-subject and within-subject variability were similar across the two conditions. This comparability offers an opportunity to identify the contributing factors, including those that may predispose individuals to greater musculoskeletal deterioration, an important consideration for the selection of future space travelers on long-duration deep-space missions.

## Supplementary information

Supplementary Information

## Data Availability

The datasets generated and/or analyzed during the current study are not publicly available due to ESA ownership of the RSL data, but are available from the corresponding author on reasonable request.

## Code availability

The custom code for supporting the conclusions of the manuscript will be made available by the corresponding author upon request.
