# Supplementary material for: Comparison of musculoskeletal responses and its variability after long-term spaceflight and prolonged bed rest conditions
Source: NPJ Microgravity. 2026 May 25;12:43. doi: 10.1038/s41526-026-00611-2 (PMC13201542; doi:10.1038/s41526-026-00611-2)
Supplement: Supplementary file 1 — Supplementary Information [file 41526_2026_611_MOESM1_ESM.docx]

**Comparison of Musculoskeletal Responses and its Variability after Long-Term Spaceflight and prolonged Bed Rest conditions**

***Supplementary Material***

***
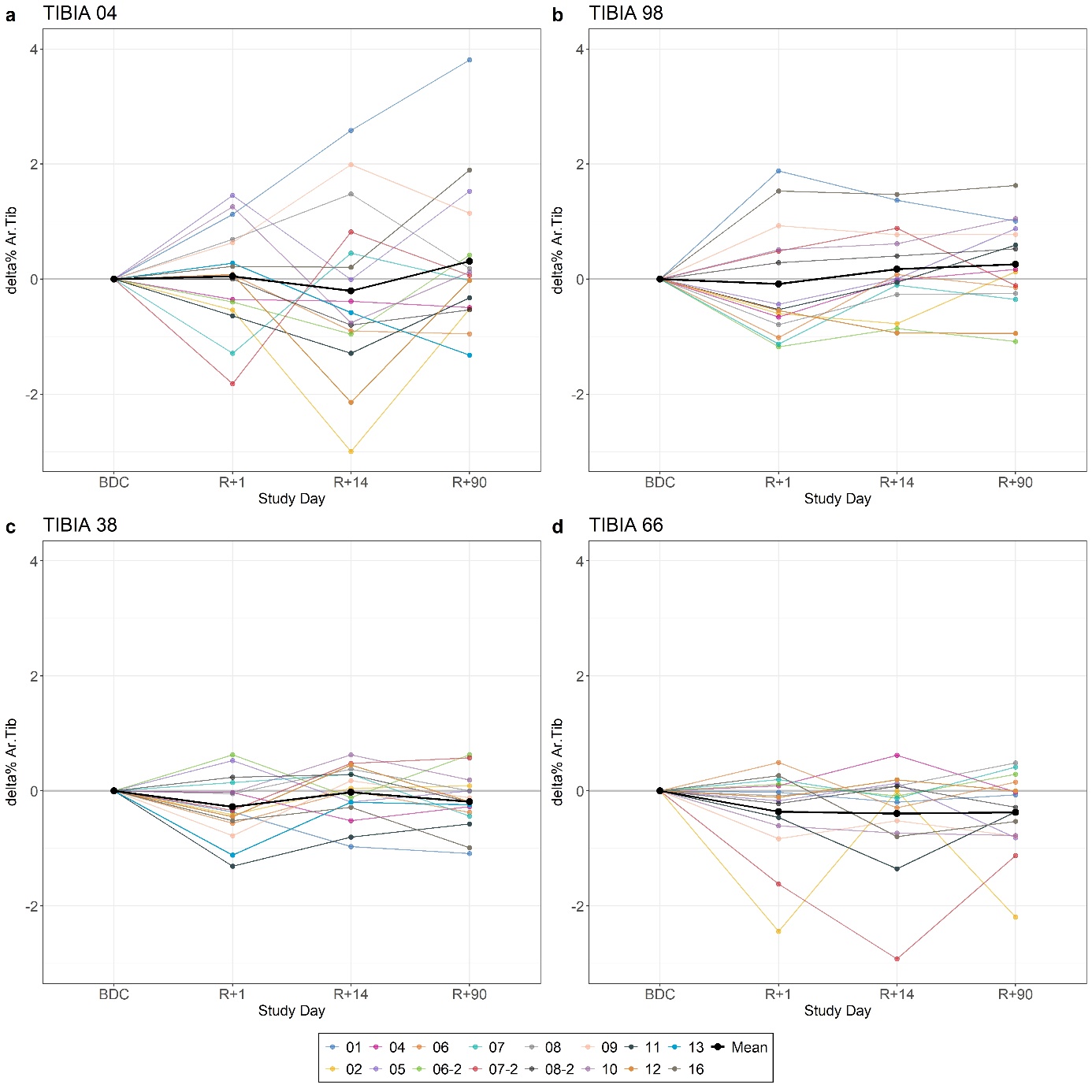
*** *Supplementary Figure 1: Percent change of Ar.Tib (bone area of the tibia) from baseline (BDC) to R+1, R+14, and R+90 after spaceflight, respectively. (a) delta% of Ar.Tib_04_, (b) delta% of Ar.Tib_98_, (c) delta% of Ar.Tib_38_, (d) delta% Ar.Tib_66_. "-02" in the legend indicates the results of the second mission of the respective space traveler. These specific results are also listed separately in Figure 2. The color indicates the different space travelers, black is the mean %delta. Statistical analysis revealed no significant differences for all measurement sites at all measurement dates.*

***
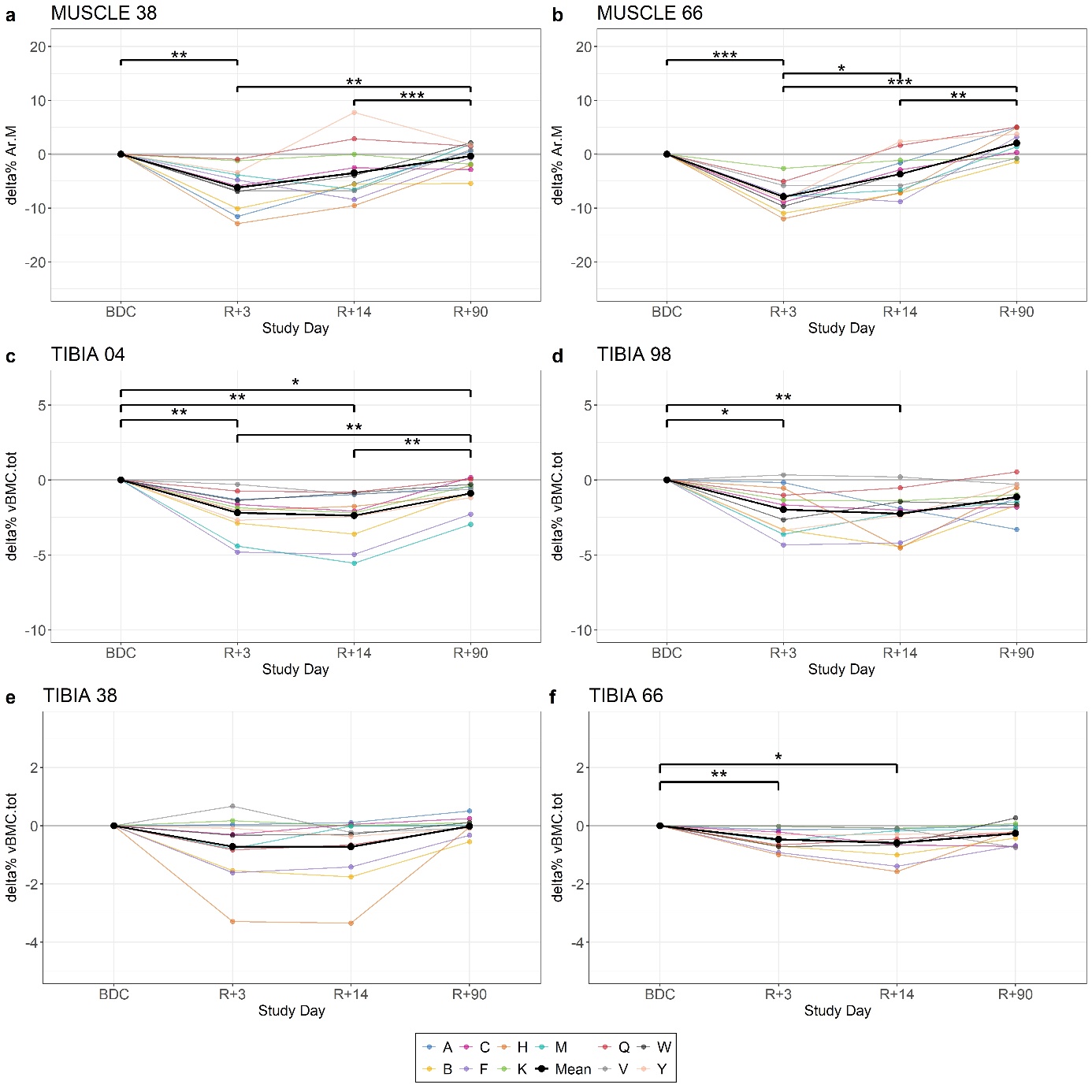
***

Supplementary Figure 2: Percent change of Ar.M and vBMC.tot from baseline (BDC) to R+3, R+14, and R+90 after bed rest, respectively. (a) delta% of Ar.M_Tib38_, (b) delta% of Ar.M_Tib66_, (c) delta% of vBMC.tot_Tib04_, (d) delta% vBMC.tot_Tib98_, (e) delta% of vBMC.tot_Tib38_, (f) delta% of vBMC.tot_Tib66_.The color indicates the different bed rest participants of the control group, which underwent bed rest only; black is the mean %delta. * significant difference with p<0.05; ** significant difference with p<0.01; *** significant difference with p<0.001.

Supplementary Table 1: Results of ANOVA with post-hoc pairwise t-test and Bonferroni adjustment comparing the several measurement dates and sites for Space. Significant differences are marked in grey. ^#^: Kruskall-Wallis-test with post-hoc Wilcoxon test and Bonferroni adjustment were performed as there was no normal distribution.

| Site | Ar.M_Tib38_ | Ar.M_Tib66_ | vBMC.tot_Tib04_ | vBMC.Ct_Tib04_^#^ | vBMC.Tb_Tib04_^#^ | vBMC.tot_Tib38_ | vBMC.Ct_Tib38_ | vBMC.tot_Tib66_ | vBMC.Ct_Tib66_ | vBMC.tot_Tib98_ | vBMC.Ct_Tib98_ | vBMC.Tb_Tib98_ |
| --- | --- | --- | --- | --- | --- | --- | --- | --- | --- | --- | --- | --- |
| BDC - R+1 | < 0.001 | < 0.001 | <0.001 | 0.002 | 1.00 | 0.01 | 0.02 | 0.04 | 0.08 | < 0.001 | 0.21 | <0.001 |
| BDC - R+14 | < 0.001 | < 0.001 | 0.003 | 0.006 | 1.00 | 0.01 | 0.014 | < 0.001 | <0.001 | < 0.001 | 0.21 | <0.001 |
| BDC - R+90 | 1.00 | 1.00 | 0.007 | 0.13 | 1.00 | 0.01 | 0.017 | 0.02 | 0.02 | 0.002 | 0.18 | 0.002 |
| R+1 - R+14 | < 0.001 | < 0.001 | 1.00 | 0.63 | 1.00 | 1.00 | 1.00 | 0.06 | 0.06 | 0.37 | 1.00 | 0.63 |
| R+1 - R+90 | < 0.001 | < 0.001 | 0.002 | 0.09 | 1.00 | 0.41 | 0.49 | 1.00 | 1.00 | 0.27 | 1.00 | 0.41 |
| R+14 - R+90 | < 0.001 | < 0.001 | 0.03 | 0.30 | 1.00 | 1.00 | 0.99 | 0.11 | 0.05 | 1.00 | 1.00 | 0.91 |
|  |  |  |  |  |  |  |  |  |  |  |  |  |

Supplementary Table 2: Results of ANOVA with post-hoc pairwise t-test and Bonferroni adjustment comparing the several measurement dates and sites for Bed Rest. Significant differences are marked in grey. ^#^: Kruskall-Wallis-test with post-hoc Wilcoxon test and Bonferroni adjustment were performed as there was no normal distribution.

| Site | | Ar.M_Tib38_ | | Ar.M_Tib66_ | | vBMC.tot_Tib04_ | | vBMC.Ct_Tib04_^#^ | | vBMC.Tb_Tib04_ | | vBMC.tot_Tib38_ | | vBMC.Ct_Tib38_ | | vBMC.Tb_Tib38_ | | vBMC.tot_Tib66_ | | vBMC.Ct_Tib66_ | | vBMC.Tb_Tib66_ | | vBMC.tot_Tib98_ | | vBMC.Ct_Tib98_^#^ | | vBMC.Tb_Tib98_ |
| --- | --- | --- | --- | --- | --- | --- | --- | --- | --- | --- | --- | --- | --- | --- | --- | --- | --- | --- | --- | --- | --- | --- | --- | --- | --- | --- | --- | --- |
| BDC - R+3 | | 0.004 | | < 0.001 | | 0.001 | | 0.74 | | 1.00 | | 0.39 | | 0.25 | | 0.04 | | 0.008 | | 0.014 | | 1.00 | | 0.01 | | 1.00 | | 0.004 |
| BDC - R+14 | | 0.23 | | 0.06 | | 0.002 | | 0.32 | | 1.00 | | 0.28 | | 0.21 | | 0.12 | | 0.03 | | 0.02 | | 1.00 | | 0.004 | | 1.00 | | 0.002 |
| BDC - R+90 | | 1.00 | | 0.12 | | 0.04 | | 1.00 | | 1.00 | | 1.00 | | 1.00 | | 1.00 | | 0.17 | | 1.00 | | 1.00 | | 0.05 | | 1.00 | | 0.03 |
| R+3 - R+14 | | 0.33 | | 0.02 | | 1.00 | | 1.00 | | 1.00 | | 1.00 | | 1.00 | | 1.00 | | 1.00 | | 0.94 | | 1.00 | | 1.00 | | 1.00 | | 1.00 |
| R+3 - R+90 | | 0.004 | | < 0.001 | | 0.001 | | 0.11 | | 1.00 | | 0.32 | | 0.19 | | 0.59 | | 1.00 | | 0.97 | | 1.00 | | 1.00 | | 1.00 | | 0.52 |
| R+14 - R+90 | | 0.34 | | 0.007 | | 0.001 | | 0.82 | | 1.00 | | 0.19 | | 0.12 | | 0.71 | | 0.55 | | 0.32 | | 1.00 | | 0.44 | | 1.00 | | 0.34 |
|  |  | |  | |  | |  | |  | |  | |  | |  | |  | |  | |  | |  | |  | |  | |

Supplementary Table 3: Results (p-values) of the Levene test comparing the variances of pc of Space and Bed Rest. Significant differences are marked in grey.

| Space and Bed Rest | | | | | | | | | | | | |
| --- | --- | --- | --- | --- | --- | --- | --- | --- | --- | --- | --- | --- |
| Site | Ar.M_Tib38_ | Ar.M_Tib66_ | vBMC.tot_Tib04_ | vBMC.Ct_Tib04_ | vBMC.Tb_Tib04_ | vBMC.tot_Tib38_ | vBMC.Ct_Tib38_ | vBMC.tot_Tib66_ | vBMC.Ct_Tib66_ | vBMC.tot_Tib98_ | vBMC.Ct_Tib98_ | vBMC.Tb_Tib98_ |
| BDC - R+1/R+3 | 0.17 | 0.03 | 0.08 | 0.83 | 0.68 | 0.96 | 0.90 | 0.14 | 0.22 | 0.08 | 0.86 | 0.06 |
| BDC - R+14 | 0.97 | 0.84 | 0.12 | 0.84 | 0.60 | 0.75 | 0.90 | 0.49 | 0.64 | 0.26 | 0.39 | 0.18 |
| BDC - R+90 | 0.24 | 0.09 | 0.11 | 0.57 | 0.70 | 0.01 | 0.08 | 0.28 | 0.78 | 0.06 | 0.29 | 0.08 |
|  |  |  |  |  |  |  |  |  |  |  |  |  |

Supplementary Table 4: Measurement Uncertainty U_Meas_ [%²] of measurements during space experiments and bed rest study. Furthermore, p-values of Wilcoxon test are presented.

| Space and Bed Rest | | | | | | |
| --- | --- | --- | --- | --- | --- | --- |
| Measurement Site | Ar.M_Tib38_ | Ar.M_Tib66_ | vBMC.tot_Tib04_ | vBMC.tot_Tib38_ | vBMC.tot_Tib66_ | vBMC.tot_Tib98_ |
| Space | 0.93 | 1.20 | 0.33 | 0.04 | 0.04 | 0.72 |
| Bed Rest | 0.56 | 1.16 | 0.13 | 0.07 | 0.01 | 0.41 |
| p | 0.83 | 0.84 | 0.10 | 0.54 | 0.15 | 0.28 |
|  |  |  |  |  |  |  |

Supplementary Table 5: Results of U_Obs_ (top) and U_IR_ (bottom) [%²] after space mission by measurement sites

| Space | | | | | | |
| --- | --- | --- | --- | --- | --- | --- |
|  | Ar.M_Tib38_ | Ar.M_Tib66_ | vBMC.tot_Tib04_ | vBMC.tot_Tib38_ | vBMC.tot_Tib66_ | vBMC.tot_Tib98_ |
| BDC - R+1 | 25.15 24.23 | 23.93 22.73 | 6.41 6.07 | 0.81 0.77 | 0.41 0.37 | 6.12 5.40 |
| BDC - R+14 | 15.65 14.73 | 14.89 13.68 | 7.86 7.53 | 0.73 0.69 | 0.41 0.37 | 5.04 4.32 |
| BDC - R+90 | 10.28 9.36 | 14.93 13.72 | 2.90 2.56 | 0.50 0.47 | 0.28 0.24 | 5.36 4.64 |
|  |  |  |  |  |  |  |

Supplementary Table 6: Results of U_Obs_ (top) and U_IR_ (bottom) [%²] after bed rest by measurement sites

| Bed Rest | | | | | | |
| --- | --- | --- | --- | --- | --- | --- |
|  | Ar.M_Tib38_ | Ar.M_Tib66_ | vBMC.tot_Tib04_ | vBMC.tot_Tib38_ | vBMC.tot_Tib66_ | vBMC.tot_Tib98_ |
| BDC - R+3 | 15.79 15.23 | 7.06 5.91 | 2.01 1.87 | 1.21 1.15 | 0.12 0.11 | 2.45 2.04 |
| BDC - R+14 | 27.05 26.49 | 14.06 12.90 | 2.75 2.62 | 1.13 1.06 | 0.28 0.27 | 2.43 2.02 |
| BDC - R+90 | 5.87 5.32 | 6.14 4.98 | 0.92 0.78 | 0.08 0.01 | 0.11 0.10 | 1.04 0.63 |
|  |  |  |  |  |  |  |
